# Obstructive rectal endometriosis treated by robot-assisted laparoscopic surgery: a case report

**DOI:** 10.1186/s40792-020-00977-9

**Published:** 2020-08-14

**Authors:** Naotaka Kuriyama, Koji Ando, Qingjiang Hu, Yu Miyashita, Yoshiaki Fujimoto, Tomoko Jogo, Kentaro Hokonohara, Ryota Nakanishi, Yuichi Hisamatsu, Yasue Kimura, Daisuke Tsurumaru, Kenichi Kohashi, Yoshinao Oda, Eiji Oki, Masataka Nishimura, Masaki Mori

**Affiliations:** 1 Department of Surgery and Science, Kyushu University, 3-1-1 Maidashi, Fukuoka, Fukuoka 812-8582 Japan; 2 Department of Anatomic Pathology, Kyushu University, 3-1-1 Maidashi, Fukuoka, Fukuoka 812-8582 Japan; 3 Department of Clinical Radiology, Kyushu University, 3-1-1 Maidashi, Fukuoka, Fukuoka 812-8582 Japan; 4 Nishimura Icho-Naika Clinic, 2-5-5 Hirao, Fukuoka, Fukuoka 810-0014 Japan

**Keywords:** Rectal endometriosis, Robot-assisted surgery, Low anterior resection, Rectal stenosis

## Abstract

**Background:**

Rectal endometriosis is a rare disease. A definitive diagnosis prior to surgery is often difficult. We encountered a patient with rectal sub-obstructive endometriosis that was treated by robot-assisted laparoscopic low anterior resection.

**Case presentation:**

A 43-year-old woman visited our hospital with suspected stenosis caused by upper rectal cancer. She had a 2-year history of constipation. We were unable to confirm the diagnosis through detailed examinations, including laparoscopy. Robot-assisted laparoscopic low anterior resection with D3 lymph node dissection was performed for both diagnosis and treatment. The postoperative specimen showed a submucosal tumor. The pathological examination confirmed rectal endometriosis.

**Conclusions:**

We herein describe a rare case of obstructive rectal endometriosis that we were unable to diagnose preoperatively. Robotic surgery was useful in this case, which involved extensive pelvic adhesion.

## Background

Endometriosis is a benign gynecological disease, and heterotopic endometriosis is rare. The most common location of heterotopic endometriosis is the gastrointestinal tract [1]. Gastrointestinal endometriosis is usually asymptomatic. It does not always show specific symptoms or imaging findings and is challenging to diagnose [2] [3]. Obstructive rectal endometriosis is rare, but when it occurs, it may be difficult to distinguish from malignant disease [4]. Definitive treatment is surgical resection, but this is challenging because of inflammation and adhesion with surrounding organs. Laparoscopic surgery has become the mainstream treatment [5], and reports of robot-assisted surgery have been increasing [6, 7]. We encountered a patient with obstructive rectal endometriosis that was difficult to distinguish from malignant disease. As far as we could search on the “Ichushi Website,” a Japanese medical database, there were no reports of robot-assisted laparoscopic surgery for rectal endometriosis in Japan. This is the first report of a Japanese patient whose rectum was safely removed from the adhered surrounding tissue by robot-assisted laparoscopic surgery.

## Case presentation

A 43-year-old woman visited our hospital with suspected stenosis caused by upper rectal cancer. She had a 2-year history of constipation with neither melena nor abdominal pain. She was nulliparous and had a stable menstrual cycle. A physical examination revealed no abnormalities. She had neither anemia nor an increased inflammatory reaction. Her carcinoembryonic antigen level was 2.5 ng/ml, and her cancer antigen 19-9 level was 31.6 U/ml.

**Fig. 1:**
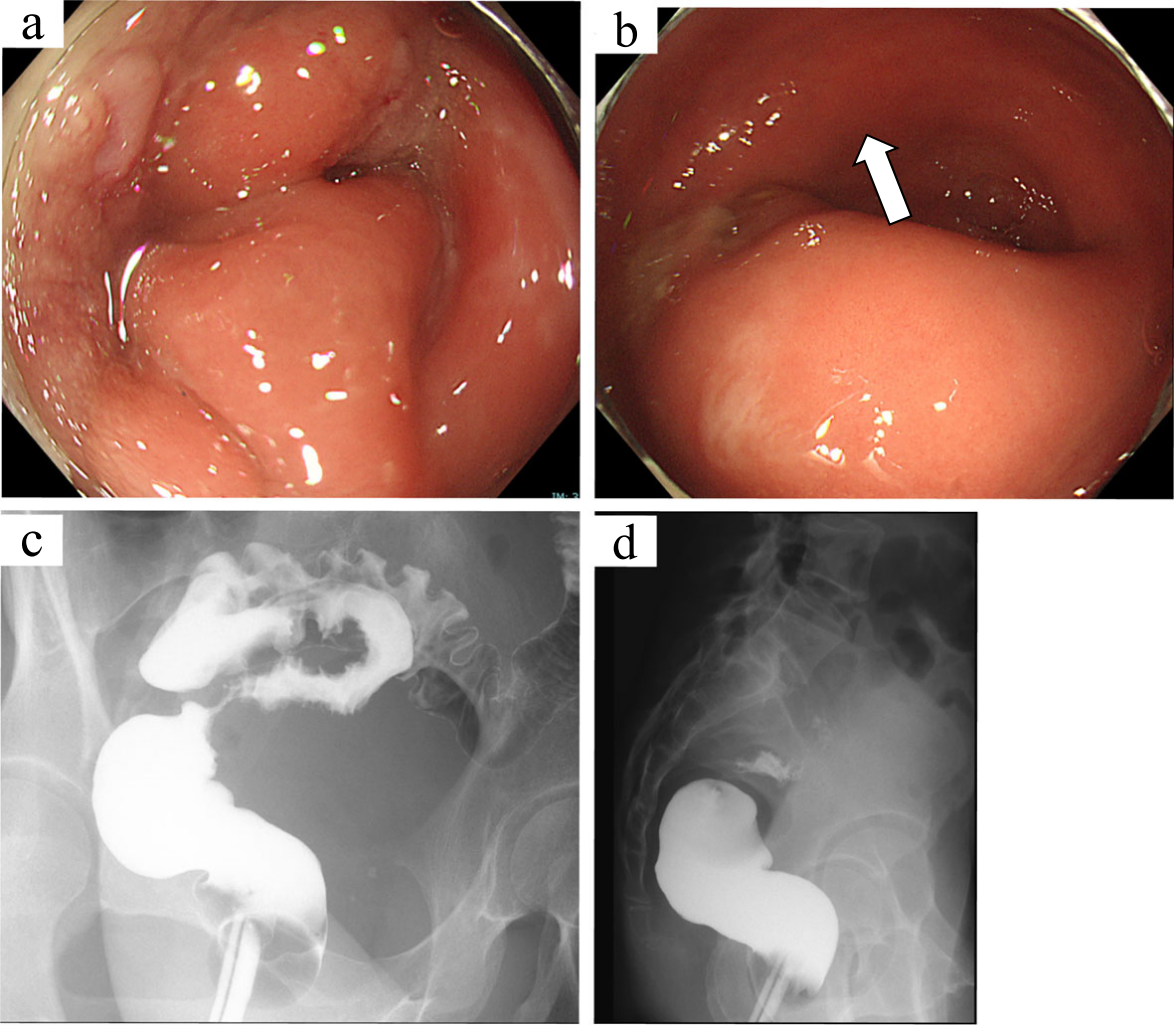
Colonoscopy and barium enema images. Colonoscopy revealed circumferential stenosis with intact and mucosa. A barium enema revealed a stenosis with a smooth surface

**Fig. 2:**
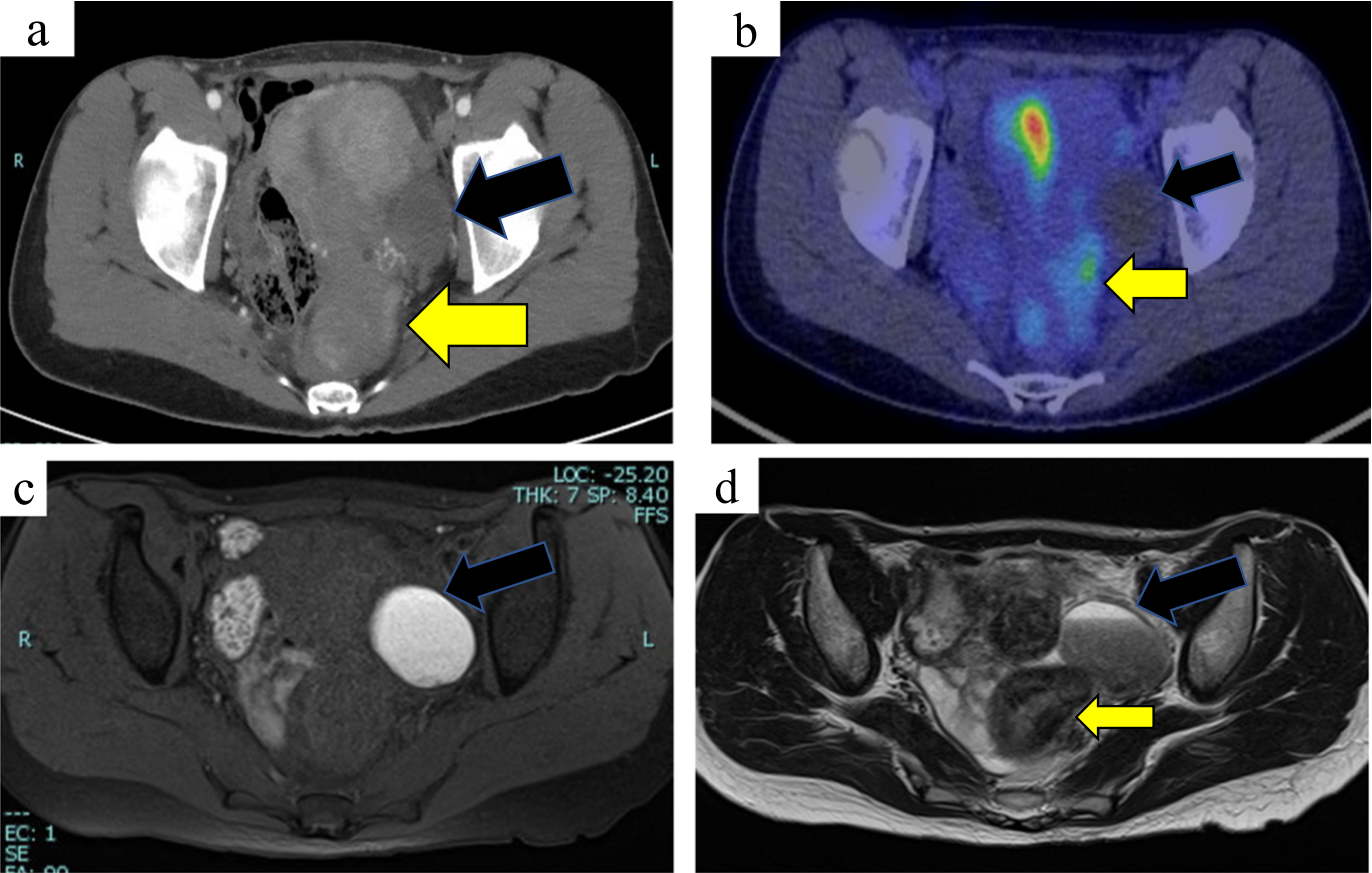
Computed tomography and magnetic resonance imaging findings. Computed tomography revealed thickening of the rectal wall (yellow arrow) and a left ovarian cyst (black arrow). Positron emission tomography-computed tomography revealed a slight increase in fluorodeoxyglucose uptake at the wall of the rectum. Magnetic resonance imaging revealed a left ovarian chocolate cyst and a thickened rectum wall

**Fig. 3:**
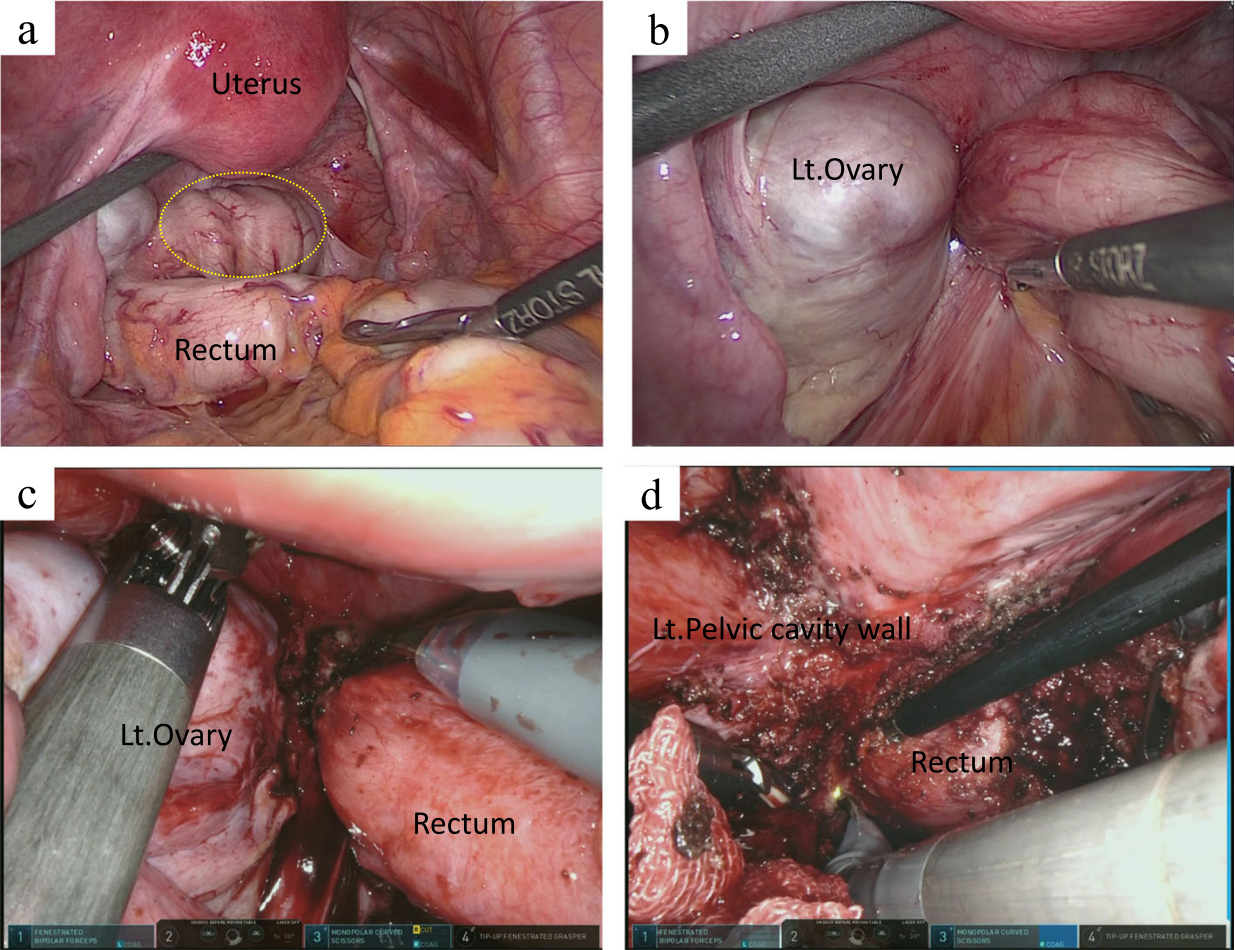
Surgical imaging. **a**, **b** An adhesion was present between the left ovary cyst and rectum. There was no evidence of endometrium tissue. **c**, **d** The adhesion was removed by robot-assisted surgery

An abdominal upright X-ray revealed a large amount of intestinal gas. Colonoscopy revealed a circumferentially elevated lesion in the upper rectum. The raised lesion extended to the inferior valve of Houston, and a normal mucous membrane covered the lesion (Fig. 1a, b). We obtained endoscopic biopsies twice, and histopathological examination revealed nonspecific inflammation. A barium enema examination revealed a stenosis with a smooth surface. The examination findings suggested the presence of an extramural mass predominantly on the left side of the stenosis (Fig. 1c, d). Contrast-enhanced computed tomography revealed wall thickening from the upper to lower rectum. There were no findings of ileus. A 4-cm cyst was found in the left ovary (Fig. 2a). Fluorodeoxyglucose (FDG)-positron emission tomography revealed a slight increase in FDG uptake in the wall of the rectum. No abnormal FDG uptake was observed in the left ovary (Fig. 2b). Magnetic resonance imaging revealed thickening of the left wall of the upper rectum (Fig. 2c, d). We performed diagnostic laparoscopy to observe the lesion directly. Neither peritoneal dissemination nor liver metastasis was observed. A small amount of ascites was observed in the pouch of Douglas. A chocolate cyst was present in the left ovary and the rectum had mild adhesions, but no endometrium was found on the rectal surface or abdominal wall. An induration was found in the upper rectum and was considered to be a tumor (Fig. 3a, b). Neither ascites cytology nor washed ascites cytology showed any malignant findings.

**Fig. 4:**
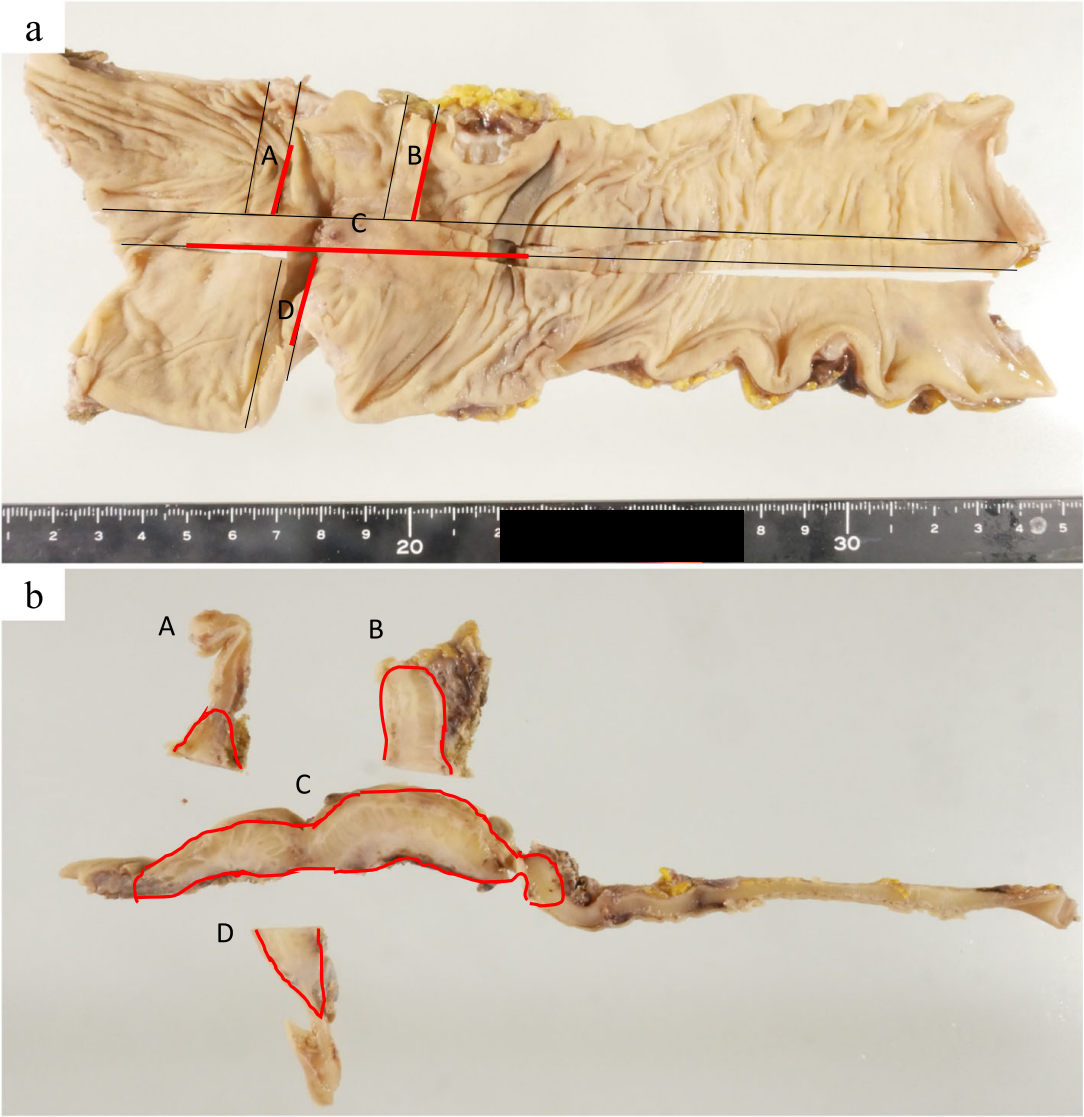
Resected specimen. The resected specimen contained an approximately 10-cm submucosal tumor-like lesion. Endometriosis showed within the red line and frame

**Fig. 5:**
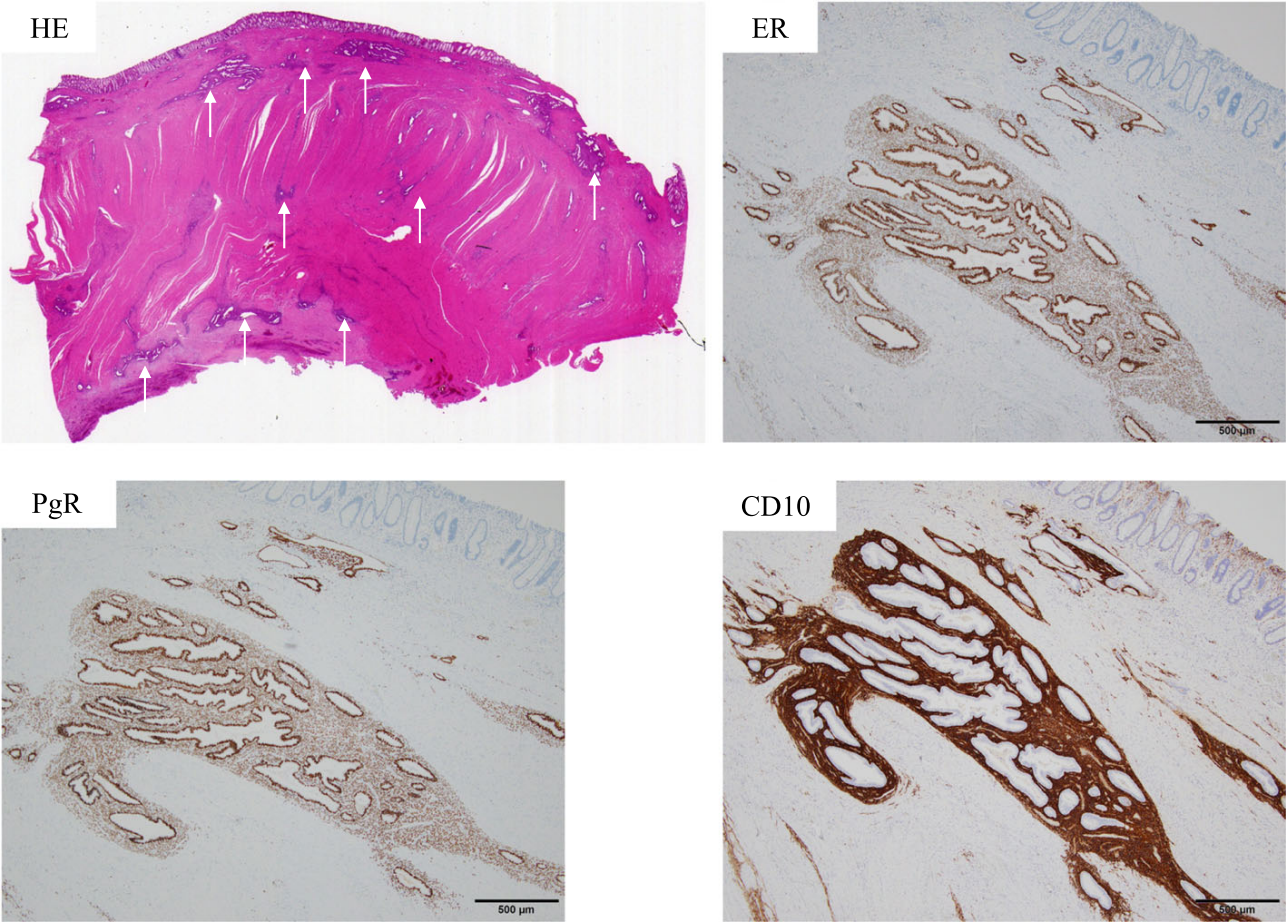
Pathological findings of the tumor. Hematoxylin and eosin staining revealed many islands of endometrial glands and stroma through the submucosa to subserosa (arrow). These tissues were positive for estrogen receptor, progesterone receptor, and cluster of differentiation 10

Because the possibility of malignant diseases such as type 4 rectal cancer or a gastrointestinal stromal tumor could not be denied and the patient desired removal of the lesion, she underwent robot-assisted laparoscopic low anterior resection with D3 lymphadenectomy. The duration of surgery was 429 min, and the blood loss was 175 ml. The left wall of the rectum was firmly adhered to the left ovary and pelvic cavity wall and was difficult to remove (Fig. 3c, d). The anastomosis was about 3 cm from the anal verge, and a large amount of stool had accumulated in the oral side of the colon. A temporary ileostomy was constructed. The patient had Clavien–Dindo grade IIIa anastomotic leakage that was treated conservatively, and she was discharged home 37 days after surgery. The temporary ileostomy was closed 3 months after surgery. The resected specimen showed a submucosal tumor. Histopathological examination revealed a 75-mm extension of thickened rectal wall with many islands of endometrial glands, and stroma was present through the submucosa to subserosa (Figs. 4 and 5). Immunohistochemically, the glandular cells were positive for estrogen receptor and progesterone receptor, and the stromal cells were positive for estrogen receptor, progesterone receptor, and cluster of differentiation 10 (Fig. 5). Rectal endometriosis was confirmed by pathological diagnosis. The chocolate cyst in the left ovary was not removed, and the patient was attending a gynecological department for hormone therapy at the time of this writing.

## Discussion

Gastrointestinal endometriosis accounts for 5 to 10% of all cases of endometriosis, and 70 to 90% of such cases occur in the sigmoid colon and rectum [8]. Symptoms of rectal endometriosis include melena, narrowing of stool, constipation, and abdominal pain, but these symptoms are not always present. Likewise, menstrual symptoms are not always present. Rectal endometriosis can cause perforation or acute bowel obstruction [9, 10].

In rectal endometriosis, severe fibrosis around the rectal wall occurs because of repeated bleeding and inflammation of the endometrial tissue in the wall. Magnetic resonance imaging findings may reflect this bleeding and inflammation [11]. The most useful diagnostic method is laparoscopy. In addition to peritoneal lesions such as blueberry spots, ectopically growing endometrial tissue can be observed on the surface of the intestinal serosa, and biopsy specimens can be obtained from this tissue. Our patient developed constipation without menstrual symptoms. Diagnostic laparoscopy revealed a chocolate cyst and adhesion between the cyst and the rectum. She had no additional findings suggesting endometriosis. Therefore, we preoperatively diagnosed malignant-like lesions such as type 4 rectal cancer or a gastrointestinal stromal tumor.

The treatment of gastrointestinal endometriosis aims to relieve symptoms and improve infertility. Medication therapy is the first choice, but it is less effective for stricture lesions. There are reports of stent placement or balloon dilation for stricture lesions, but they eventually required surgery. These treatments are the aspects of the bridge to surgery [12]. Surgical treatment is selected when melena or an obstruction is present or when symptom relief with medical therapy is difficult [13]. Because intestinal endometriosis is a benign disease, excessively invasive therapy should be avoided. Laparoscopic surgery has historically been performed, and robot-assisted laparoscopic surgery has been recently reported in an effort to decrease surgical invasiveness. In patients with gastrointestinal endometriosis, especially rectal endometriosis, the ovary, uterus, and deep pelvis are often simultaneously affected by endometrial tissue. Deep pelvic endometriosis may be close to or involved in the ureter and pelvic plexus. Injury to these organs can cause serious complications. The advantages of laparoscopic surgery include good operability in a narrow space and a magnifying effect. The advantages of robot-assisted surgery include stability of the operative field and free movement of forceps, in addition to the advantages of laparoscopic surgery. Our patient had a chocolate cyst in the left ovary, the adhesion between the left pelvic wall and rectum was strong, and adhesive delamination was complicated [14, 15]. Especially in the case of adhesive delamination around the peritoneal reflection, the robot-assisted surgery showed a higher advantage in the stability of the operative field and the operability of the forceps compared with the standard laparoscopic surgery.

At our institution, since this surgical procedure was covered by insurance in 2018, we have actively applied this procedure for case of upper rectum and lower rectum disease after giving sufficient informed consent. Then, we performed robot-assisted laparoscopic low anterior resection with D3 lymphadenectomy. In rectal endometriosis, lesions may be found in lymph nodes. Some reports have linked this pathological significance to the malignant transformation of the lesions, but the details are unknown and malignant transformation is very rare. Therefore, lymphadenectomy is not always required unless a malignant disease is suspected [16]. The extent of stenosis was estimated to be 2 to 3 cm in the upper rectum by barium enema (Fig. 1c, d). Intraoperative findings showed that the adhesion had strongly spread from the area of the stenosis to the anus. In the resected specimen, submucosal tumor-like lesions extended for about 10 cm, and endometriotic lesions were present in the broad area of the resected specimen (Fig. 4). These widespread lesions might have resulted in the rectal stenosis. Because of the chocolate cyst and possible residual intestinal endometriosis, we considered that hormonal therapy was necessary to prevent recurrence after surgery [16].

## Conclusions

In summary, we experienced a rare case of rectal endometriosis that we could not diagnose preoperatively. We performed robot-assisted laparoscopic low anterior resection in this case. This procedure is considered very useful for cases with strong adhesions and fibrosis due to inflammation and is an example of expanding the possibility of robot-assisted surgery in Japan.

## Data Availability

Data sharing is not applicable to this article, as no datasets were generated or analyzed during the current study.

## Abbreviations

FDG: Fluorodeoxyglucose
